# *Leishmania* and phlebovirus seroprevalence in dogs in two non-endemic central European countries: Austria and Germany

**DOI:** 10.1016/j.onehlt.2026.101580

**Published:** 2026-09-13

**Authors:** Katharina Platzgummer, Majda Globokar Vrhovec, Nikola Pantchev, Johanna Krug, Karin Deinhammer, Gioia Bongiorno, Aldo Scalone, Maria Sophia Unterköfler, Hans-Peter Fuehrer, Markus Milchram, Sandra Oerther, Julia Walochnik, Elif Kurum, Nazli Ayhan, Remi Charrel, Edwin Kniha

**Affiliations:** a Institute of Specific Prophylaxis and Tropical Medicine, Center for Pathophysiology, Infectiology and Immunology, Medical University of Vienna, Vienna, Austria; b IDEXX Laboratories, Kornwestheim, Germany; c Small Animal Practice Weissbach, Steina, Germany; d Small Animal Practice Tierärzteteam, Neunkirchen, Austria; e Vector-borne Diseases Unit, Istituto Superiore di Sanità, Rome, Italy; f Center of Pathobiology, Institute of Parasitology, University of Veterinary Medicine Vienna, Vienna, Austria; g Institute of Zoology, Department of Ecosystem Management, Climate and Biodiversity, BOKU University, Vienna, Austria; h Coordination Center for Bat Conservation and Bat Research Austria, Leonding, Austria; i Independent Researcher, Karlsdorf-Neuthard, Germany; j Unité des Virus Émergents (UVE), Aix-Marseille Univ, Università di Corsica, IRD 190, Inserm 1207, IRBA, Marseille, France; k National Reference Center for Arboviruses, Inserm-IRBA, Marseille, France

**Keywords:** Canine leishmaniosis, IFAT, *Leishmania infantum*, Sand fly, Toscana virus, Sandfly fever Sicilian virus

## Abstract

In Mediterranean Europe, human and canine leishmanioses, mainly caused by the sand fly-borne protozoan parasite *Leishmania infantum*, are highly endemic, and Phlebovirus infections are common. Dogs represent the main reservoir hosts of *L. infantum*, facilitating zoonotic vector-borne circulation. Although phleboviruses are primarily of medical importance in humans, dogs are regularly exposed due to the overlapping ecology of *Leishmania* and phleboviruses and serve as good sentinels for circulation.

This study aimed to assess canine *Leishmania* and phlebovirus seroprevalence in two understudied non-endemic Central European countries, Austria and Germany.

1520 dog sera from Austria (*n* = 760) and Germany (*n* = 760) were screened for anti-*Leishmania* antibodies by immunofluorescence antibody tests (IFAT) and for anti-phlebovirus antibodies using seroneutralization assays. For positive tested dogs of Austrian or German origin without travel history, clinical, anamnestic data, and, if available, PCR-based confirmation was assessed.

*Leishmania* seroprevalence was 0.8% (95% CI: 0.3–1.7%) in Austrian and 1.2% (95% CI: 0.5–2.2%) in German dogs. Phlebovirus seroprevalence was 1.7% (95% CI: 0.9–2.9%) and 2.9% (95% CI: 1.8–4.4%) in these study populations, respectively. No statistically significant difference in seropositivity for both agents was observed between dogs living in sand fly endemic versus non-endemic regions. While most *Leishmania*-seropositive dogs had been imported from *Leishmania*-endemic countries, three had been born in Austria or Germany and had never travelled to endemic regions.

Our study highlights that seropositive dogs living in non-endemic countries are mainly imported from endemic regions. Nevertheless, few potential local transmission events highlight the complexity of sand fly-borne disease epidemiology and require broader surveillance programs.

## Highlights

• *Leishmania* seropositive dogs living in Austria and Germany are mostly imported from endemic countries.
• Few suspected local transmissions highlight the need for broader surveillance programs.
• Regions with sand fly presence may promote autochthonous zoonotic transmission of *Leishmania infantum*.
• A need for increased surveillance of human and canine leishmaniasis in non-endemic countries is highlighted.

## 1 Introduction

From a One Health perspective, canine vector-borne diseases (CVBDs) are of high public health relevance due to the close contact between dogs and humans, and the frequent exposure of dogs to vector-borne pathogens of veterinary and medical importance [1]. In Mediterranean Europe, the zoonotic sand fly-borne protozoan parasites *Leishmania* spp. (Kinetoplastida: Trypanosomatidae) and phleboviruses are highly prevalent [2], [3]. Considering global warming, sand fly-borne diseases are expected to spread northwards to previously non-endemic regions, including Central Europe [4].

Although a variety of domestic and wild animals, including rodents, hares, and carnivores have been found to be susceptible to *Leishmania* spp. infections, dogs are the main reservoir hosts of *Leishmania infantum*, zoonotically transmitted to humans by various vector species. Very importantly, clinically asymptomatic infected dogs may be infective to sand flies, leading to an underestimation of its true circulation, and complicating epidemiological assessments. Following a potentially long incubation period, the onset of diverse clinical signs might be delayed for months to even years after infection [5]. In general, canine leishmaniosis (CanL) is a systemic disease often affecting multiple organs. Although skin lesions are the most common clinical manifestation, involvement of internal organs poses the greatest threat to the dog's health, with glomerulonephritis being the most common cause of death [6].

Another group of sand fly-borne pathogens (SFBPs) endemic in Mediterranean Europe are phleboviruses. Of particular importance are Sandfly fever Sicilian virus (SFSV; *Phlebovirus siciliaense*) and Toscana virus (TOSV, *Phlebovirus toscanaense*), which can cause a febrile illness in humans, also known as three-day fever or sand fly fever. Noteworthy, TOSV can be neuroinvasive, causing meningitis and meningoencephalitis [7]. Unlike humans, dogs do not appear to develop disease after infection. However, cases of co-infections with phleboviruses and *Leishmania* have been reported in humans as well as dogs [8], [9], and evidence from experimental murine models suggests that phlebovirus co-infection may exacerbate clinical signs of leishmaniosis and increase the risk of relapse by altering the host's innate immune responses, including enhanced inflammatory signaling that promotes parasite survival [10], [11]. While dogs are likely not involved in maintaining phlebovirus transmission cycles, CanL might promote vector-host transmission of phleboviruses [12]. In addition, phlebovirus seroprevalence may reflect exposure to sand flies, and thus the circulation of SFBPs in a certain region [13], making dogs good sentinels for local transmission.

Noteworthy, alternative routes of *Leishmania* transmission have been described, including blood transfusions, vertical transmission from an infected female to her pups, and venereal transmission from male to female dogs [14], [15]. Transmission during dog fights has also been documented [16], [17]. These transmission routes should not be disregarded, particularly since there is no specific immune prophylaxis available against phleboviruses and two vaccines against *L. infantum* available for dogs in Europe reduce parasite burden and disease severity, but do not prevent infection or provide sterilizing immunity. Consequently, a multimodal preventive approach is necessary, including the reduction of exposure through the use of registered ectoparasiticides with repellent activity to protect from sand fly bites as well as immunomodulators with proven efficacy [6], [18].

Particularly, in non-endemic countries such as Austria and Germany, where infections with SFBPs are not notifiable, data on human and canine leishmanioses and phlebovirus infections are scarce. To date, only few suspected autochthonous *Leishmania* infections in humans and dogs with unclear transmission routes have been reported from the two countries [19], [20], [21], [22], [23], and only a single study suggested local TOSV transmission in Germany [24]. In Austria, the only human TOSV cases are recorded from returning travelers from endemic countries [25]. Moreover, the predominant sand fly species in both countries is *Phlebotomus mascittii*, a species with general low abundance [26], but strongly indicated vector capacity for *L. infantum* under laboratory conditions [27]*.*

The most important source of *L. infantum* introduction into non-endemic regions are imported dogs from endemic countries and travel of dogs to endemic countries with their owners, most importantly the Mediterranean. Antibodies against *L. infantum* have been found in 12.2% to 23.5% of mostly imported or travelling dogs, indicating frequent exposure [23], [28], [29], although the risk of infection with arthropod-borne pathogens decreases with shorter travels [30], [31].

Therefore, the increasing importation of dogs from countries endemic for SFBPs [32], together with the observed presence and anticipated expansion of sand flies in Austria and Germany due to climatic changes [26], underscores the need to evaluate the current epidemiological changes of SFBPs such as *L. infantum* and phleboviruses in dogs residing in these countries. This is particularly relevant in sand fly-endemic regions, where autochthonous transmission to other dogs but also humans could theoretically exist but remains insufficiently studied. Therefore, the aim of the present study was to assess the seroprevalence of SFBPs in dogs living in Austria and Germany, two currently non-endemic countries.

## 2 Material & methods

### 2.1 Sample collection and random selection of dog sera

Between November 2023 and October 2025, a total of 2471 dog sera submitted by veterinarians to a private diagnostic laboratory for routine diagnostic purposes were collected, of which 959 originated from Austrian dogs and 1512 from German dogs. Samples were obtained for various medical reasons and examined with the agreement of the owners. Anonymized data included information on age and sex of dogs, and postal codes of the submitting veterinary practices. For dogs that tested positive for at least one of the pathogens investigated in the present study, the respective veterinarians were contacted for information regarding the dog’'s country of origin and travel activities.

For the selection of 1520 dog sera (760 from Austria and Germany, respectively, following sample size calculations of the CLIMOS project, https://climos-project.eu/), postal codes of all samples were assigned to bioregions of Austria [33] and Germany (“Naturräume”) [34]. These bioregions were additionally categorized in sand fly endemic and non-endemic based on available literature [26]. Subsequently, all sera from sand fly-endemic bioregions were selected. For non-endemic sand fly bioregions, we used a stratified random sample of 760 sera per country, with bioregions serving as strata. Sampling was performed using the *ssamp* command from the R package *sampler* [35]. In Austria, 533 (70.1%) dog sera were available from sand fly-endemic bioregions, whereas in Germany, 198 of the sera (26.1%) originated from dogs living in sand fly-endemic bioregions.

Altogether, 1520 dogs were tested for antibodies against *Leishmania*, SFSV, and TOSV. Among the 760 dogs from Austria, 507 (66.7%) were female and 249 (32.8%) were male; for four (0.5%) dogs, information on sex was not available. The age of the dogs ranged from <1 to 21 years (mean = 9.3 years). Among the 760 dogs from Germany, 421 (55.4%) were female and 335 (44.1%) were male; for four (0.5%) dogs, information on sex was not available. The age of the dogs ranged from <1 to 18 years (mean = 8.8 years).

### 2.2 *Leishmania* screening using Immunofluorescent Antibody Tests (IFAT) and qPCR

Serum samples were screened for IgG antibodies against *Leishmania* using a commercial indirect Immunofluorescent Antibody Tests (IFAT) (MegaFLUO® LEISH, MEGACOR Diagnostik GmbH, Hörbranz, Austria) with initial dilutions of 1:100 (cut-off value provided by manufacturer). A sensitivity of 99.4% and specificity of 92% is indicated by the manufacturer. Positive controls (ready-to-use provided by the manufacturer) as well as negative controls were always added. All positive sera were sent to a partner laboratory to confirm positivity and determine titers by two-fold dilution series using an in-house IFAT (sensitivity: 99.0%, specificity: 98.0%) using antigen from *L. infantum* culture promastigotes (WHO reference strain MHOM/TN/1980/IPT-1) and Anti-Dog IgG-FITC conjugate (Sigma #F4012) diluted 1:200 in PBS, with serum dilutions of 1:80 considered as positive threshold antibody titer [36]. Positive controls (serum from a dog with a parasitologically confirmed diagnosis) and negative controls were always added.

For cases with potential local transmission, DNA extracted from skin and blood samples retrospectively provided by the diagnostic laboratory were screened for *Leishmania* spp. with a probe-based qPCR protocol targeting the kinetoplast DNA (kDNA) using primers and probe from Mary et al. [37] and using the Luna® Universal Probe qPCR Master Mix (New England Biolabs, Ipswich, MA, USA) with a final volume of 20 μL and a Bio-Rad CFX96 Touch Real-Time PCR Detection System (Bio-Rad Laboratories, Inc., Hercules, CA, USA) with an initial cycle of 95 °C for 3 min followed by 45 cycles each consisting of 95 °C for 15 s and 60 °C for 1 min. *Leishmania infantum* DNA corresponding to 10.2 parasites/mL was used as positive control.

### 2.3 Phlebovirus screening using seroneutralization assays (SNA)

All dog sera were heat-inactivated at 56 °C for 30 min, then two-fold dilutions from 1/20 to 1/160 of each serum sample were tested for anti-TOSV and anti-SFSV antibodies. The serum dilution was mixed in equal volumes with viral suspension of 100 TCID₅₀ of TOSV (Origin: France, Strain: 5904242804, EVA strain: UVE/TOSV/2014/FR/5904, EVA Ref-SKU: 001 V-02452, Genbank Acc: KU935736, https://www.ncbi.nlm.nih.gov/nuccore/KU935736) and SFSV (Origin: Italy, Strain: Sabin, EVA strain: UVE/SFSV/1943/IT/Sabin, EVA Ref-SKU: 001v-EVA77, Genbank Acc: EF095551, https://www.ncbi.nlm.nih.gov/nuccore/EF095551) in 96-well microplates. Following a 1-h incubation at 37 °C, 100 μL of a Vero E6 cell suspension (5 × 10^5^ cells/mL) was added to each well using epMotion 5075 (Eppendorf, Hamburg, Germany). Each plate included positive control (cells with virus) and negative controls (the first serum dilution with cells and only cells) and each batch of test included TCID_50_ assay for viral titration confirmation. After 5 days for TOSV and 6 days for SFSV, photos of each well of the 96-well microplates were taken by using Incucyte SX5 Live-Cell Analysis (Sartorius, Göttingen, Germany) and results were analyzed to determine cytopathic effect (CPE) and neutralization titers. A sensitivity of 98.1% and specificity of 98.8% is calculated for CPE-based virus neutralization test (VNT) with reference to PRNT90 for Zika virus [38]. Neutralizing antibodies titers ≥1/40 were considered as positive to avoid false positive results. All positive samples were re-tested for confirmation.

### 2.4 Statistical analysis and mapping

Seroprevalence and the corresponding 95% confidence intervals (CI) were calculated using the exact binomial method for each different pathogen, stratified by age groups according to Harvey [39], sex and bioregions (sand fly-endemic vs. non-endemic). Due to small numbers of events and separation detected using the R package *detectseparation* [40], differences between the groups were assessed using Firth's bias-reduced logistic regression to obtain unbiased estimates, with models fitted in R using the package *logistf* [41], and model fit was evaluated by examining predicted probabilities. *P*-values of <0.05 were considered significant. All statistical analyses were performed using R version 4.5.2 [42].

Seropositive dogs were mapped using the central coordinates of postal codes. To estimate numbers of *Leishmania* and phlebovirus seropositive dogs by bioregion, we accessed recent available online data on estimated dog population sizes of 836,000 in Austria (https://www.statista.com/statistics/515489/dog-population-europe-austria/) and 10,500,000 in Germany (https://www.statista.com/statistics/515489/dog-population-europe-germany/). Thereafter, human population data [43] were intersected with bioregions to derive estimated number of humans per bioregion. Consequently, we calculated dog density proportional to human population density by biogeographic region:

$$D_{i}=D_{\mathrm{total}}\times \frac{H_{i}}{H_{\mathrm{total}}}$$

where:

$D_{i}$= is the estimated number of dogs in biogeographic region *i*

$D_{\mathrm{total}}$ = is the total number of dogs in the study area

$H_{i}$ = is the human population in biogeographic region *i*

$H_{\mathrm{total}}$ = is the total human population across all biogeographic regions

Observed overall seroprevalence rates of *Leishmania* and phleboviruses by country were then applied to calculate seropositivity and 95% CIs using Wilson intervals. Estimates were mapped through color-coding maps of bioregions in QGIS [44].

## 3 Results

### 3.1 *Leishmania* seroprevalence

An overall *Leishmania* seroprevalence of 0.8% (6/760; 95% CI: 0.3–1.7) and 1.2% (9/760; 95% CI: 0.5–2.2) was detected in Austrian and German dogs, respectively. Antibody titers of positive samples ranged from 1:80 to 1:640 in Austrian dogs and from 1:80 to 1:2560 in German dogs (Table 1).

**Table 1:** Immunofluorescent Antibody Test (IFAT)-based *Leishmania* seroprevalence in tested Austrian and German dogs.

*Leishmania* IFAT titers	Austrian dogs (*n* = 760)		German dogs (n = 760)	
	positive	prevalence (95% CI)	positive	prevalence (95% CI)
1:80	4	0.5% (0.1–1.3)	2	0.3% (0.0–0.9)
1:160	0	–	1	0.1% (0.0–0.7)
1:320	1	0.1% (0.0–0.7)	1	0.1% (0.0–0.7)
1:640	1	0.1% (0.0–0.7)	1	0.1% (0.0–0.7)
1:1280	0	–	2	0.3% (0.0–0.9)
1:2560	0	–	2	0.3% (0.0–0.9)
Total	6	0.8% (0.3–1.7)	9	1.2% (0.5–2.2)

### 3.2 Phlebovirus seroprevalence

An overall phlebovirus seroprevalence of 1.7% (13/760; 95% CI: 0.9–2.9) and 2.9% (22/760; 95% CI: 1.8–4.4) was detected in Austrian and German dogs, respectively.

In Austria, 11 (1.5%; 95% CI: 0.7–2.6) dogs were SFSV seropositive and 2 (0.3%; 95% CI: 0.0–0.9) were TOSV seropositive (Table 2). One sample was positive for both anti-TOSV and anti-*Leishmania* antibodies.

**Table 2:** Seroneutralization assay (SNA)-based SFSV and TOSV seroprevalence in tested Austrian dogs.

Titers	SFSV seroneutralization (n = 760)		TOSV seroneutralization (n = 760)	
	Positive	Prevalence (95% CI)	Positive	Prevalence (95% CI)
1:40	10	1.3% (0.6–2.4)	2	0.3% (0.0–0.9)
1:80	1	0.1% (0.0–0.7)	0	–
Total	11	1.4% (0.7–2.6)	2	0.3% (0.0–0.9)

In Germany, 20 (2.6%; 95% CI: 1.6–4.0) dogs were SFSV seropositive and 2 (0.3%; 95% CI: 0–0.9) were TOSV seropositive (Table 3). One sample was positive for both anti-SFSV and anti-*Leishmania* antibodies.

**Table 3:** Seroneutralization assay (SNA)-based SFSV and TOSV seroprevalence in tested German dogs.

Titers	SFSV seroneutralization (n = 760)		TOSV seroneutralization (n = 760)	
	Positive	Prevalence (95% CI)	Positive	Prevalence (95% CI)
1:40	18	2.4% (1.4–3.7)	2	0.3% (0.0–0.9)
1:80	2	0.3% (0.0–0.9)	0	–
Total	20	2.6% (1.6–4.0)	2	0.3% (0.0–0.9)

### 3.3 Seroprevalences by sex, age group, and bioregion

**Fig. 1:**
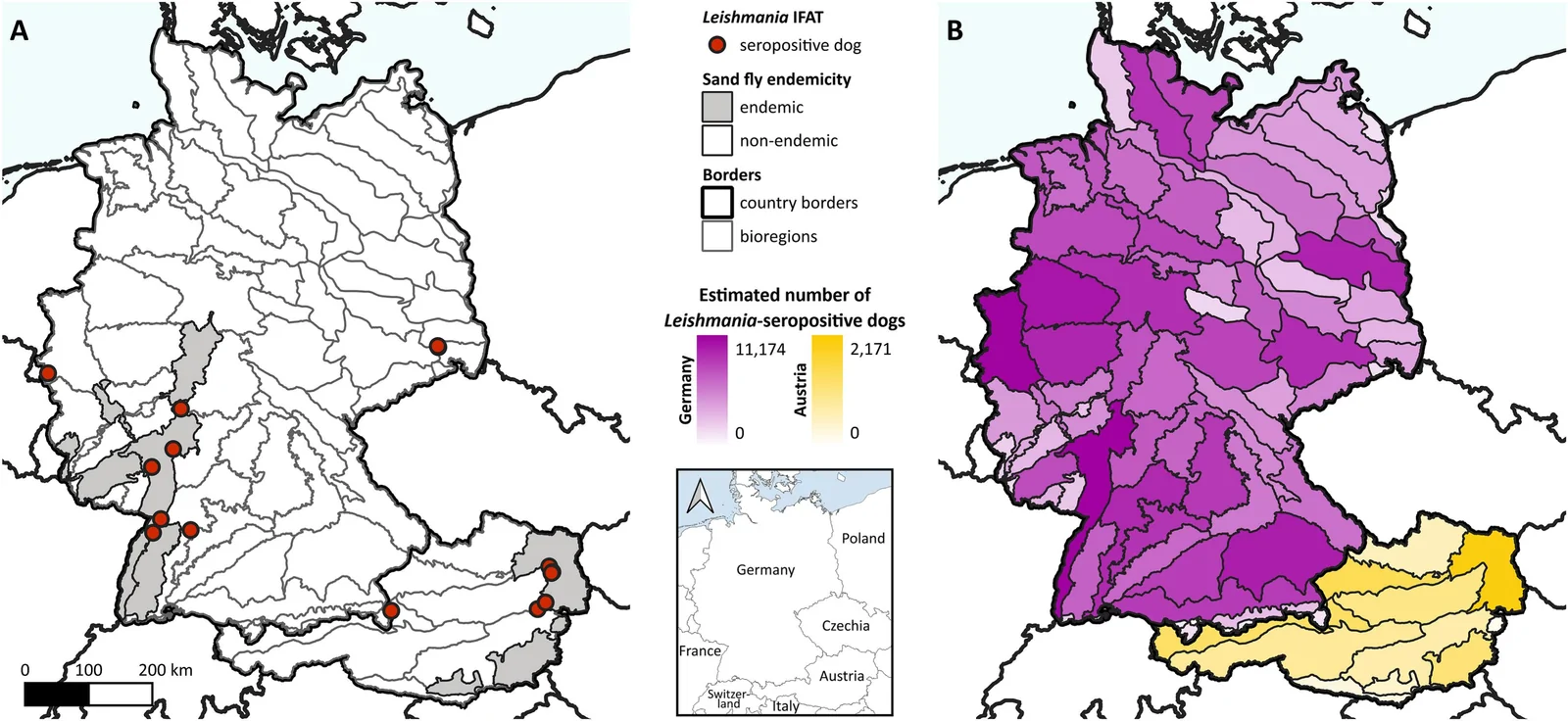
*Leishmania* seroprevalence in dogs from Austria and Germany. Geographic distribution of seropositive dogs and known sand fly endemicity by bioregion in Austria and Germany (A). Seroprevalence-based estimated number of anti-*Leishmania* antibody positive dogs per bioregion (B). Centralized zip codes were used as location of anti-*Leishmania* antibody positive dogs.

**Fig. 2:**
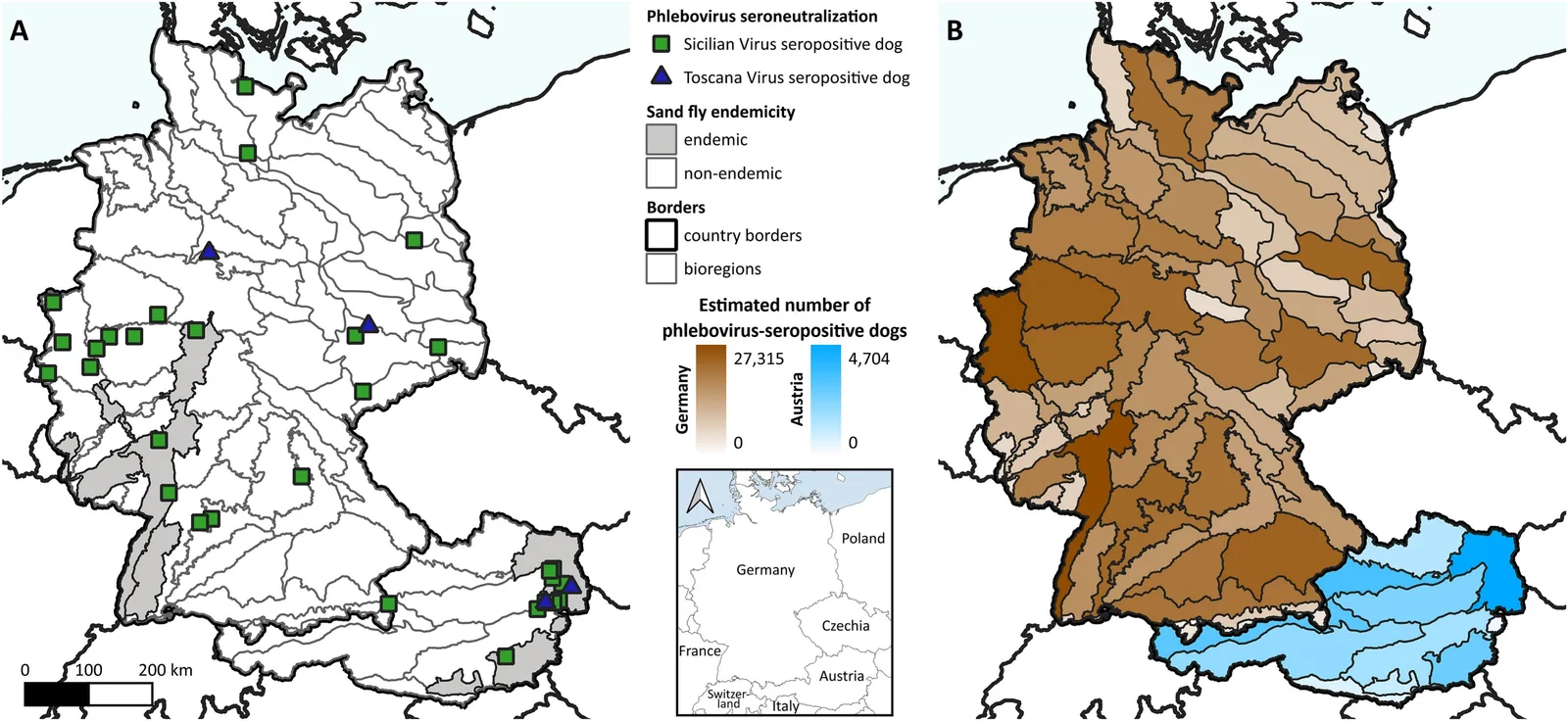
Phlebovirus (TOSV and SFSV) seroprevalence in dogs from Austria and Germany. Geographic distribution of seropositive dogs and known sand fly endemicity by bioregion in Austria and Germany (A). Seroprevalence-based estimated number of anti-Phlebovirus antibody (TOSV and SFSV) positive dogs per bioregion (B). Centralized zip codes were used as location of anti-*Leishmania* antibody positive dogs.

In Austria, *Leishmania* seroprevalence was higher in male dogs (1.6%) than in females (0.4%), whereas seroprevalence for both SFSV and TOSV was not significantly lower in males (Table 4). The highest *Leishmania* seroprevalence (1.7%) was detected in dogs younger than three years, while highest seroprevalences of SFSV (3.6%) and TOSV (0.9%) were observed in dogs aged 3–6 (Table 4). *Leishmania* seroprevalence was similar in sand fly-endemic and non-endemic bioregions (0.8% vs. 0.9%). SFSV and TOSV seroprevalences were non-significantly higher in sand fly-endemic regions (Table 4, Fig. 1A, Fig. 2A).

**Table 4:** *Leishmania,* SFSV, and TOSV seroprevalences of Austrian dogs by age group, sex, and biogeographic region.

	n Dogs	*Leishmania* IFAT		SFSV Seroneutralization		TOSV Seroneutralization	
Factor		Positive	Prevalence (95% CI)	Positive	Prevalence (95% CI)	Positive	Prevalence (95% CI)
Age							
<3	58	1	1.7% (0.0–9.2)	1	1.7% (0.0–9.2)	0	–
3–6	112	0	–	4	3.6% (1.0–8.9)	1	0.9% (0.0–4.9)
7–9	186	3	1.6% (0.3–4.6)	1	0.5% (0.0–3.0)	1	0.5% (0.0–3.0)
10–11	154	0	–	1	0.6% (0.0–3.6)	0	–
12–14	206	2	1.0% (0.1–3.5)	3	1.5% (0.3–4.2)	0	–
>14	44	0	–	1	2.3% (0.1–12.0)	0	–

Sex							
Male	249	4	1.6% (0.4–4.1)	3	1.2% (0.2–3.5)	0	–
Female	507	2	0.4% (0.0–1.4)	8	1.6% (0.7–3.1)	2	0.4% (0.0–1.4)
n/a	4	0	–	0	–	0	–

Region^a^							
Present	533	4	0.8% (0.2–1.9)	10	1.9% (0.9–3.4)	2	0.4% (0.0–1.3)
Absent	227	2	0.9% (0.1–3.1)	1	0.4% (0.0–2.4)	0	–
Total	760	6	0.8% (0.3–1.7)	11	1.5% (0.7–2.6)	2	0.3% (0.0–0.9)

a present = sand fly-endemic bioregions, absent = non-endemic bioregions.

In Germany, *Leishmania* seroprevalence was significantly higher in male dogs (2.1%) compared to females (0.5%). Also, males showed a higher SFSV seroprevalence, but lower TOSV seroprevalence, both not significantly (Table 5). The highest *Leishmania* seroprevalence (3.2%) was observed in dogs in the age class 10–11, while highest SFSV (4.8%) and TOSV (1.5%) seroprevalences were observed in dogs aged 10–11 and < 3, respectively (Table 5). *Leishmania* seroprevalence was higher in sand fly-endemic bioregions (2.5%) compared to non-endemic (0.7%), whereas SFSV and TOSV seroprevalences were lower in sand fly-endemic bioregions, although differences were not statistically significant (Table 5, Fig. 1A, Fig. 2A).

**Table 5:** *Leishmania,* SFSV, and TOSV seroprevalences of German dogs by age group and sex*,* and biogeographic region.

	n Dogs	*Leishmania* IFAT		SFSV Seroneutralization		TOSV Seroneutralization	
Factor		Positive	Prevalence (95% CI)	Positive	Prevalence (95% CI)	Positive	Prevalence (95% CI)
Age group							
<3	67	1	1.5% (0.0–8.0)	2	3% (0.4–10.4)	1	1.5% (0.0–8.0)
3–6	145	3	2.1% (0.4–5.9)	0	–	0	–
7–9	181	1	0.6% (0.0–3.0)	6	3.3% (1.2–7.1)	0	–
10–11	125	4	3.2% (0.9–8.0)	6	4.8% (1.8–10.2)	1	0.8% (0.0–4.4)
12–14	198	0	–	6	3.0% (1.1–6.5)	0	–
>14	44	0	–	0	–	0	–

Sex							
Male	335	7	2.1% (0.8–4.3)^b^	10	3.0% (1.4–5.4)	0	–
Female	421	2	0.5% (0.1–1.7)^b^	10	2.4% (1.1–4.3)	2	0.5% (0.1–1.7)
n/a	4	0	–	0	–		

Region^a^							
Present	198	5	2.5% (0.8–5.8)	4	2.0% (0.6–5.1)	0	–
Absent	562	4	0.7% (0.2–1.8)	16	2.8% (1.6–4.6)	2	0.4% (0.0–1.3)
Total	760	9	1.2% (0.5–2.2)	20	2.6% (1.6–4.0)	2	0.3% (0.0–0.9)

a present = sand fly-endemic bioregions, absent = non-endemic bioregions.

b *p*-value<0.05.

### 3.4 Estimated numbers of *Leishmania* and phlebovirus positivity

The overall estimated number of *Leishmania* seropositive dogs based on mean prevalences of 0.8% and 1.2% was 6599 (95% CI: 3028–14,310) in Austria and 121,343 (95% CI: 65,534–234,690) in Germany, respectively. By bioregion, estimates ranged from 0 to 2171 specimens in Austria and 0 to 11,174 in Germany, respectively. Overall, 3098 (95% CI: 1421–6718) seropositive dogs in Austria and 17,209 (95% CI: 9071–32,483) in Germany were estimated to live in sand fly-endemic bioregions (Fig. 1B).

Based on mean phlebovirus seroprevalences of 1.7% in Austria and 2.9% in Germany, overall numbers were estimated to be 14,301 (95% CI: 8378–24,281) in Austria and 303,946 (95% CI: 201,525–456,122) in Germany, respectively. Estimates of seropositive dogs by bioregion ranged from 1 to 4704 specimens in Austria and 0 to 27,315 specimens in Germany, respectively. The highest estimated counts were found in Eastern Austria and Southwest Germany, corresponding to sand fly-endemic regions (Fig. 1B, Fig. 2B). All estimated numbers and 95% CIs by bioregion are available in Supplementary Table 1 and Supplementary Table 2.

### 3.5 Follow-up of seropositive dogs

The country of origin and travel history could be determined for 14 of 15 dogs tested positive for anti-*Leishmania* antibodies (Table 6, Table 7). Six countries of origin were identified, seven dogs originated from Spain, two from Italy, one from Bosnia and Herzegovina (BIH) and Türkiye, respectively. Two dogs from Austria and one dog from Germany had no travel history to *Leishmania*-endemic countries (Table 6, Table 7). The three dogs with a SFSV titer of 1:80 were of Slovakian, German and unknown origin respectively, the latter dog had travelled to Croatia (Supplementary Table 3).

Medical histories for two of the three *Leishmania* seropositive dogs from Austria and Germany without travel history were provided by the respective veterinarians.

**Table 6:** Details of dogs from Austria positive for anti-*Leishmania* antibodies.

IFAT Titer	TOSV/SFSV Positive (Titer)	Age	Sex	Place of Birth	Known Travel History	Clinical Picture
80	no	13	male	unknown	unknown	n/a
80	no	7	female	Austria	Germany, Netherlands	various symptoms
80	TOSV (1/40)	8	female	Spain	unknown	n/a
80	no	12	male	Austria	none	symptoms typical for leishmaniosis
320	no	9	male	Spain	unknown	infection known and treated
640	no	2	male	Italy	unknown	blood abnormalities matching leishmaniosis

**Table 7:** Details of dogs from Germany positive for anti-*Leishmania* antibodies.

IFAT Titer	TOSV/SFSV Positive (Titer)	Age	Sex	Place of Birth	Known Travel History	Clinical Picture and Symptoms
80	no	3	male	Italy	unknown	infection known
80	no	10	male	Spain	unknown	n/a
160	SFSV (1/40)	11	male	Spain	unknown	inconspicuous
320	no	11	male	Spain	unknown	inconspicuous
640	no	3	male	Türkiye	unknown	inconspicuous
1280	no	8	female	Spain	unknown	inconspicuous
1280	no	4	female	BIH	unknown	infection known
2560	no	2	male	Spain	unknown	diarrhea, blood abnormalities
2560	no	11	male	Germany	Germany	symptoms typical for leishmaniosis

The Austrian dog was a 12-year-old male Chihuahua with persistent inflammatory symptoms including stomatogingivitis and disseminated hair loss, which persisted for approximately one year after the positive IFAT (low antibody titer of 1:80) in the present study, despite symptomatic, immunosuppressive, and antibiotic treatment (Table 6). Histological examination of a skin sample taken at that time showed a periadnexal chronic mononuclear plasmacytosis with pigmentary incontinence with no pathogens detected although leishmanial etiology would be compatible with this inflammatory pattern. qPCR-based screening for *Leishmania* DNA was negative. A *Leishmania*-specific ELISA performed in a veterinary laboratory (Afosa test according to Wolf et al. [45]) was also negative one year after the positive IFAT result. The dog was born and had lived exclusively in Austria, although the dog's parental travel history could not be determined.

**Supplementary Fig. 1:**
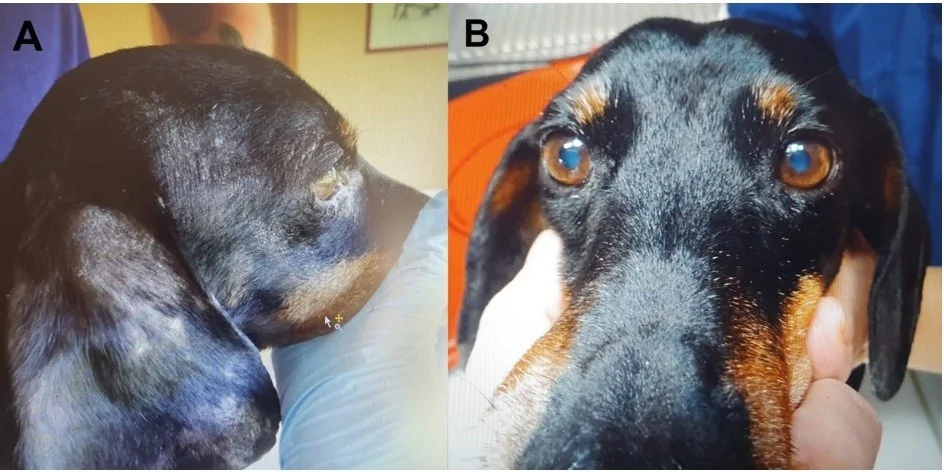
Clinical signs of canine leishmaniosis in a German wirehaired dachshund. Before treatment, showing alopecic skin lesions and exfoliation around the eyes (A). After treatment, with improvement of the lesions and hair regrowth (B). Photo credit: J. Krug.

The German dog was an 11-year-old male wirehaired dachshund with an onset of cutaneous symptoms such as hair loss and exfoliation around the eyes (Table 7, Supplementary Fig. 1). A *Leishmania*-specific ELISA (Afosa test according to Wolf et al. [45]) was performed by a veterinary laboratory, yielding a highly positive result of 96 test units (positive cutoff of 12 test units) along with increased total serum proteins (increased beta-2 globulins and polyclonal gamma globulinemia), based on which combination treatment with miltefosine and allopurinol (according LeishVet.org guidelines) was started. This led to complete clinical remission with decline in ELISA antibody levels to 51 test units within three months and normalization of laboratory abnormalities. The IFAT test performed in the present study, using a serum sample taken approximately three months after beginning of treatment, showed a high antibody titer (1:2560) and a *Leishmania*-qPCR from blood was positive (C_T_ = 36.3). The dog was born in Bavaria and had never travelled outside Germany and the owners confirmed that also the dog's parents had never travelled abroad. The dog had no history of dog fights or breeding activity.

## 4 Discussion

The present study reports novel data on *Leishmania* and phlebovirus seroprevalence in dogs from Austria and Germany, providing insights into the epidemiology of these pathogens in two non-endemic Central European countries. Our results highlight the critical role of importation of seropositive dogs from endemic regions, potentially introducing CanL into non-endemic countries. Based on the observed prevalence rates, we aimed to present a first estimate on the number of seropositive dogs living in both countries.

Previous studies in Germany and Austria focused primarily on screening imported or travelling dogs for antibodies against *Leishmania*, reporting seroprevalence rates ranging from 3.6% to over 20% [23], [28], [30], [32]. We analyzed a less-biased random selection of dog sera samples submitted to laboratories for routine diagnostics. This approach revealed prevalence rates of 0.8% in Austria and 1.2% in Germany. While data from non-endemic countries remain scarce, these findings align well with expected lower seroprevalence rates in dogs compared to endemic countries. Similarly to our study, Kotnik et al. [46] reported an estimated seroprevalence of 1.9% in dogs from non-endemic Slovenia, whereas higher rates were reported from endemic countries by comparable studies in Kosovo (3.5%) [9], Portugal (12.5%) [47], or Italy (weighted mean prevalence 23.3%) [48], however, with notable regional variations.

Contrary to a regularly reported increase of seroprevalence with age in endemic countries [49], we did not observe a significant age-related trend in our study. A sex-related seroprevalence, as previously reported in some studies [23], [50], was observed only in German dogs. This may be partly attributed to a limited statistical power of our sample size, or might reflect patterns seen in studies focusing on imported or travelling *Leishmania*-positive dogs in non-endemic countries [29], [31].

Dogs as the main reservoir hosts of *L. infantum* [51] are increasingly imported from endemic countries to Central Europe or accompany their owners on travels [31], [32]. Most of the *Leishmania* seropositive dogs in our study were imported from Spain, Italy, BIH, and Türkiye, generally showing low antibody titers indicating previous exposure rather than active infection. We did, however, notice a clustering of dogs seropositive for anti-*Leishmania* antibodies in sand fly-endemic bioregions [26], which also represent the most densely populated areas of Austria and Germany [43]. Two Austrian dogs with low antibody levels and one German dog with high antibody levels and qPCR positivity did not have any travel history, which highlights the potential of local infection. While both Austrian dogs originate from sand fly-endemic bioregions, the German dog is living in a non-endemic region for sand flies and, as its parents, was born in the federal state of Bavaria (Germany), where previous studies detected antibodies against *L. infantum* in a dog [23] as well as in sheep [52]. Autochthonous transmission has been assumed, although no sand flies have been recorded in Bavaria to date, despite targeted field studies [53].

Currently, local transmission of *Leishmania* involving dogs and sand flies is likely rare due to the small-scale distribution of sand flies in Central Europe [26]. Therefore, alternative transmission routes should be considered, including direct (e.g., fighting/biting), vertical, or venereal transmission from males to females, as well as blood transfusion [16], [54]. Notably, in Germany, a dog was infected with *Leishmania* through wounds after biting a *Leishmania*-positive dog imported from Spain [17]. In Czechia, transplacental transmission has been reported across three consecutive generations [55]. It is therefore crucial to raise awareness among veterinarians and breeders that exposure to *Leishmania* parasites can be possible even in Central European countries [6].

Despite limitations, using laboratory-submitted samples, uneven regional representation, assumptions linking dog density to human population density, and relatively small numbers of positive cases, we estimated pathogen seroprevalence across bioregions in both countries. We extrapolated that up to 3098 dogs in Austria and 17,208 dogs in Germany could be living in sand fly-endemic bioregions, urging further large-scale studies. However, these calculations may overstate the epidemiological representativeness of the calculated estimates, and should be considered as a first general assessment. Importantly, seropositivity does not reflect active infection, but exposure. Thus, inference on local transmission dynamics is impossible without further clinical assessment. Additional factors such as parasitemia levels in dogs affecting their infectivity to sand flies and vector presence must also be taken into account [56].

For the first time, we present data on phlebovirus seroprevalence in dogs from both Austria and Germany. Our results show a markedly higher SFSV seroprevalence compared to TOSV, consistent with findings from endemic countries [7], [8]. No co-exposure to both viruses was observed, but one German dog tested positive for both anti-SFSV and anti-*Leishmania* antibodies, and one dog from Austria had antibodies against both TOSV and *Leishmania*. While seroprevalence rates are lower compared to those reported in endemic Mediterranean countries, where values range from 3.8% to 60.2% [7], [8], [13], [57], [58], [59], they are notably high for two non-endemic countries.

Although only low antibody titers were detected in all positive dogs, the high prevalence, particularly for SFSV, indicates frequent import or travel to endemic countries. One of the SFSV-seropositive dogs had been imported from non-endemic Slovakia, and another one frequently travelled to Croatia with its owners. Noteworthy, a SFSV-seropositive dog from Germany had never been to an endemic country and is not living in a sand fly-endemic region. This further highlights the need for sand fly surveys in areas that have not been included in entomological field studies to date.

While we present completely novel baseline data for two non-endemic countries, this study shows some limitations. A follow-up examination of all positive dogs is lacking, as it was not our focus, thus, crucial data on seroconversion is not available. Although treatment does not clear infection and dogs remain a possible reservoir even after decline of symptoms, treating clinical dogs decreases antibody levels [60], [61].

In addition to serological testing, more sensitive PCR-based screenings (e.g., in mucosal swabs of the conjunctiva) should be applied to confirm active infection of seropositive dogs or subclinical infection of seronegative dogs. Importantly, the screening of samples submitted by veterinarians for routine diagnostics may have introduced a bias to this study, as owners who bring their dogs for regular check-ups are more likely to use prophylactic measures against vector-borne diseases, such as insect repellents. This could lead to an underestimation of the true number of dogs exposed to SFBPs. The possibility of subclinical infections in dogs, the influence of clinical signs and treatment on pathogen transmission as well as the risk of overlooking non-endemic diseases during routine veterinary visits are important factors that influence the likelihood of further SFBPs transmission and the establishment of local transmission cycles, and should therefore be considered in future studies.

## 5 Conclusion

Our findings of anti-*Leishmania* and phlebovirus antibodies suggest that dogs in Austria and Germany are exposed to SFBPs, typically associated with imports or travel to Mediterranean endemic regions. Few seropositive dogs without travel history indicate a potential for sporadic local transmission, but a full disease profile and history are required for confirmation. The study highlights the importance of testing and preventive measures to mitigate the risk of parasitic diseases being imported from endemic countries. Considering the presence of sand flies in parts of Central Europe, future One Health studies should integrate animal and human (sero)prevalence data with environmental factors that may facilitate the establishment of local transmission cycles.

The following are the supplementary data related to this article.

Supplementary Table 1 Estimated number of *Leishmania* and phlebovirus seropositive dogs by biogeographic region in Austria based on observed seroprevalence rates in this study.

Supplementary Table 2 Estimated number of *Leishmania* and phlebovirus seropositive dogs by biogeographic region in Germany based on observed seroprevalence rates in this study.

Supplementary Table 3 Detailed presentation of dogs with anti-SFSV antibodies with a titre of 1/80.

## CRediT authorship contribution statement

**Katharina Platzgummer:** Writing – review & editing, Writing – original draft, Visualization, Methodology, Formal analysis, Conceptualization. **Majda Globokar Vrhovec:** Writing – review & editing, Resources, Data curation. **Nikola Pantchev:** Writing – review & editing, Resources, Data curation. **Johanna Krug:** Writing – review & editing, Resources, Data curation. **Karin Deinhammer:** Writing – review & editing, Resources, Data curation. **Gioia Bongiorno:** Writing – review & editing, Resources, Methodology, Formal analysis. **Aldo Scalone:** Writing – review & editing, Resources, Methodology, Formal analysis. **Maria Sophia Unterköfler:** Writing – review & editing, Resources, Methodology, Formal analysis. **Hans-Peter Fuehrer:** Writing – review & editing, Resources, Methodology, Formal analysis. **Markus Milchram:** Writing – review & editing, Validation, Formal analysis, Conceptualization. **Sandra Oerther:** Writing – review & editing, Investigation, Formal analysis. **Julia Walochnik:** Writing – review & editing, Validation, Resources, Conceptualization. **Elif Kurum:** Writing – review & editing, Resources, Methodology, Formal analysis. **Nazli Ayhan:** Writing – review & editing, Resources, Methodology, Formal analysis. **Remi Charrel:** Writing – review & editing, Resources, Methodology, Formal analysis. **Edwin Kniha:** Writing – review & editing, Writing – original draft, Visualization, Supervision, Project administration, Funding acquisition, Formal analysis, Conceptualization.

## Ethical approval

Not applicable. Only anonymized remnants of dog sera submitted by veterinarians to a private diagnostic laboratory for routine diagnostic purposes were used in this study. Follow-up information was collected and provided with owners' consent.

## Ethics declaration

### Animal subject

This study was conducted in accordance with the ARRIVE (Animal Research: Reporting of In Vivo Experiments) guidelines.

Ethics committee approval was not required. Only anonymized remnants of dog sera submitted by veterinarians to a private diagnostic laboratory for routine diagnostic purposes were used in this study. Follow-up information was collected and provided with owners' consent.

## Funding

This study is co-funded by 10.13039/501100000780European Commission grant 101057690 and 10.13039/100014013UKRI grants 10038150 and 10039289, and is catalogued by the CLIMOS Scientific Committee as CLIMOS number 050 (http://www.climos-project.eu). The contents of this publication are the sole responsibility of the authors and do not necessarily reflect the views of the 10.13039/501100000780European Commission, the Health and Digital Executive Agency, or 10.13039/100014013UKRI. Neither the European Union nor granting authority nor UKRI can be held responsible. The funders had no role in study design, data collection and analysis, decision to publish, or preparation of the manuscript. For the purposes of Open Access, the authors have applied a CC BY [option: CC BY-ND] public copyright license to any Author Accepted Manuscript version arising from this submission. The six Horizon Europe projects, BlueAdapt, CATALYSE, CLIMOS, HIGH Horizons, IDAlert, and TRIGGER, form the Climate Change and Health Cluster. The Study Abroad Postgraduate Education Scholarship (MEB 1416), awarded by the Ministry of National Education of the Republic of Türkiye, is acknowledged for supporting Elif Kurum's postgraduate fellowship.

## Declaration of competing interest

The authors declare that they have no known competing financial interests or personal relationships that could have appeared to influence the work reported in this paper.

## Data Availability

All data are available in the manuscript and the supplementary files.
