# Supplementary material for: Leishmania and phlebovirus seroprevalence in dogs in two non-endemic central European countries: Austria and Germany
Source: One Health. 2026 Sep 13;23:101580. doi: 10.1016/j.onehlt.2026.101580 (PMC13601367; doi:10.1016/j.onehlt.2026.101580)
Supplement: Supplementary file 1 [file mmc4.docx]

**Supplementary Table 3.** Detailed presentation of dogs with anti-SFSV antibodies with a titre of 1/80.

| **country** | **voucher** | **titre** | **co-exposure** | **age** | **sex** | **origin** | **travel** | **clinical picture** |
| --- | --- | --- | --- | --- | --- | --- | --- | --- |
| **Austria** | **AT** | 80 | none | 12 | female | Slovakia | none | na |
| **Germany** | **DE134** | 80 | none | 9 | female | Germany | none | na |
| **Germany** | **DE252** | 80 | none | 11 | male | unknown | Croatia | na |
